# Supplementary material for: Clinical-Pathological Characteristics of Adenosquamous Esophageal Carcinoma: A Propensity-Score-Matching Study
Source: J Pers Med. 2023 Mar 3;13(3):468. doi: 10.3390/jpm13030468 (PMC10057829; doi:10.3390/jpm13030468)
Supplement: Supplementary file 1 [file jpm-13-00468-s001.zip › jpm-2233500-supplementary.pdf]

**Supplementary Figure S1.** Survival curves of EC with three different histological classifications in the raw data.

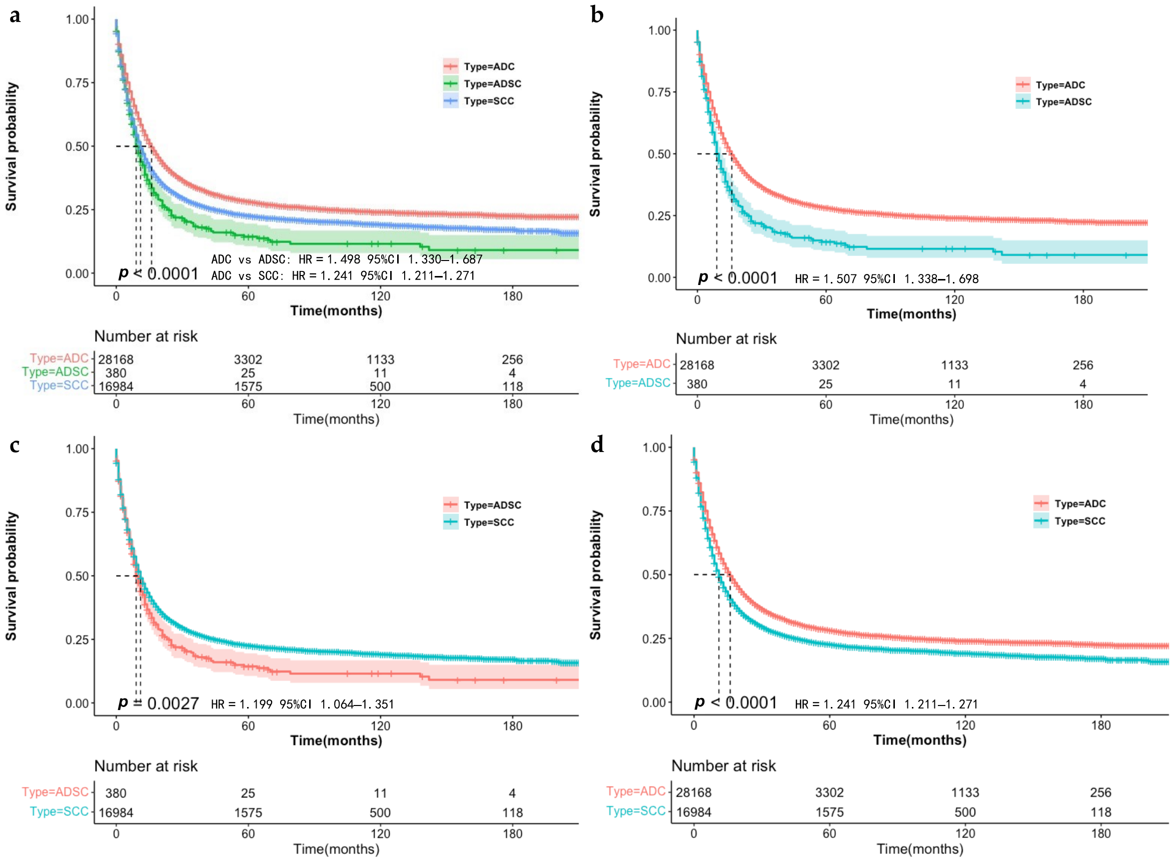


**Supplementary Table S1.** Univariable and multivariable Cox proportion hazard regression analyses for mortality in 380 ADSC patients.

| Variables | Univariable analysis | | | Multivariable analysis | | |
|---|---|---|---|---|---|---|
| | HR | 95% CI | *P* | HR | 95% CI | *P* |
| Sex | | | | | | |
| Female | 1 | reference | | 1 | reference | |
| Male | 0.895 | 0.670–1.195 | 0.451 | 1.070 | 0.784–1.461 | 0.670 |
| Age | | | | | | |
| <65 | 1 | reference | | 1 | reference | |
| >64 | 1.083 | 0.854–1.373 | 0.513 | 0.938 | 0.727–1.209 | 0.619 |
| Surgery | | | | | | |
| None | 1 | reference | | 1 | reference | |
| Surgical resection | 0.377 | 0.281–0.505 | 0.000 | 0.380 | 0.271–0.532 | 0.000 |
| Unknown | 0.944 | 0.399–2.354 | 0.903 | 0.713 | 0.269–1.886 | 0.495 |
| Radiotherapy | | | | | | |
| No | 1 | reference | | 1 | reference | |
| Yes | 0.565 | 0.443–0.719 | 0.000 | 0.719 | 0.545–0.947 | 0.019 |
| Unknown | 0.301 | 0.111–0.818 | 0.018 | 0.238 | 0.084–0.647 | 0.007 |
| Chemotherapy | | | | | | |
| No | 1 | reference | | 1 | reference | |
| Yes | 0.539 | 0.421–0.690 | 0.000 | 0.556 | 0.416–0.744 | 0.000 |
| Marital status | | | | | | |

| | | | | | | |
|---|---|---|---|---|---|---|
| Unmarried | 1 | reference | | 1 | reference | |
| Married | 0.723 | 0.567–0.922 | 0.009 | 0.887 | 0.682–1.154 | 0.372 |
| Unknown | 1.318 | 0.690–2.517 | 0.403 | 1.759 | 0.852–3.632 | 0.127 |
| Grade | | | | | | |
| I-II | 1 | reference | | 1 | reference | |
| III-IV | 1.346 | 0.924–1.960 | 0.122 | 1.314 | 0.889–1.943 | 0.171 |
| Unknown | 1.879 | 1.215–2.908 | 0.005 | 1.511 | 0.958–2.382 | 0.076 |
| Location | | | | | | |
| Upper thoracic esophagus | 1 | reference | | 1 | reference | |
| Middle thoracic esophagus | 0.743 | 0.366–1.506 | 0.410 | 0.962 | 0.460–2.011 | 0.917 |
| Lower thoracic esophagus | 0.668 | 0.341–1.308 | 0.239 | 1.045 | 0.515–2.119 | 0.903 |
| The position of thoracic esophagus is unknown | 0.866 | 0.428–1.752 | 0.689 | 1.152 | 0.554–2.394 | 0.705 |
| T | | | | | | |
| T1 | 1 | reference | | 1 | reference | |
| T2 | 0.941 | 0.489–1.810 | 0.856 | 1.327 | 0.671–2.625 | 0.415 |
| T3 | 0.670 | 0.436–1.029 | 0.068 | 0.849 | 0.539–1.338 | 0.481 |
| T4 | 1.587 | 0.966–2.606 | 0.068 | 1.312 | 0.770–2.235 | 0.317 |
| Tx | 1.288 | 1.894–1.856 | 0.174 | 1.112 | 0.696–1.777 | 0.658 |
| N | | | | | | |
| N0 | 1 | reference | | 1 | reference | |
| N1 | 0.922 | 0.672–1.266 | 0.617 | 1.063 | 0.754–1.500 | 0.726 |
| N2 | 1.167 | 1.861–1.583 | 0.319 | 1.585 | 0.881–2.853 | 0.125 |
| M | | | | | | |
| M0 | 1 | reference | | 1 | reference | |
| M1 | 2.268 | 1.672–3.078 | 0.000 | 1.402 | 0.957–2.054 | 0.083 |
| Mx | 1.525 | 1.145–2.032 | 0.004 | 0.826 | 0.466–1.466 | 0.514 |
